# An improved ShuffleNetV2 method based on ensemble self-distillation for tomato leaf diseases recognition

**DOI:** 10.3389/fpls.2024.1521008

**Published:** 2025-01-21

**Authors:** Shuiping Ni, Yue Jia, Mingfu Zhu, Yizhe Zhang, Wendi Wang, Shangxin Liu, Yawei Chen

**Affiliations:** ^1^ School of Computer Science and Technology, Henan Polytechnic University, Jiaozuo, China; ^2^ Research and Development Department, Henan Chuitian Technology Corporation Limited, Hebi, China

**Keywords:** tomato leaf diseases recognition, lightweight model, ShuffleNetV2, ensemble, self-distillation, model compression

## Abstract

**Introduction:**

Timely and accurate recognition of tomato diseases is crucial for improving tomato yield. While large deep learning models can achieve high-precision disease recognition, these models often have a large number of parameters, making them difficult to deploy on edge devices. To address this issue, this study proposes an ensemble self-distillation method and applies it to the lightweight model ShuffleNetV2.

**Methods:**

Specifically, based on the architecture of ShuffleNetV2, multiple shallow models at different depths are constructed to establish a distillation framework. Based on the fused feature map that integrates the intermediate feature maps of ShuffleNetV2 and shallow models, a depthwise separable convolution layer is introduced to further extract more effective feature information. This method ensures that the intermediate features from each model are fully preserved to the ensemble model, thereby improving the overall performance of the ensemble model. The ensemble model, acting as the teacher, dynamically transfers knowledge to ShuffleNetV2 and the shallow models during training, significantly enhancing the performance of ShuffleNetV2 without changing the original structure.

**Results:**

Experimental results show that the optimized ShuffleNetV2 achieves an accuracy of 95.08%, precision of 94.58%, recall of 94.55%, and an F1 score of 94.54% on the test set, surpassing large models such as VGG16 and ResNet18. Among lightweight models, it has the smallest parameter count and the highest recognition accuracy.

**Discussion:**

The results demonstrate that the optimized ShuffleNetV2 is more suitable for deployment on edge devices for real-time tomato disease detection. Additionally, multiple shallow models achieve varying degrees of compression for ShuffleNetV2, providing flexibility for model deployment.

## 1 Introduction

In 2022, the global tomato cultivation area was approximately 4.92 million hectares, yielding around 186.11 million tons of produce. However, tomato yields continue to be adversely affected by factors such as climate conditions and pest infestations (Wang et al., 2024). Various diseases can affect tomato plants at different growth stages, hindering their growth and ultimately leading to reduced yield and lower quality (Liang and Jiang, 2023). Traditional methods for identifying tomato diseases rely on manual inspection, a process that is both time-consuming and labor-intensive (Pandiyaraju et al., 2024). With the development of computer vision and deep learning technologies, these manual approaches have been increasingly replaced by automated solutions. However, high-performance models often come with a large number of parameters, which makes them difficult to deploy efficiently on edge devices. This poses a significant challenge for large-scale, real-time detection of tomato diseases (Zhou et al., 2024). Therefore, developing a more compact model that delivers performance comparable to larger models is essential for achieving both real-time and accurate disease detection in tomatoes.

Methods for identifying leaf diseases based on computer vision are divided into two categories: machine learning methods and deep learning methods. In terms of machine learning (Qin et al., 2016), applied a segmentation method combining the K-median clustering algorithm with linear discriminant analysis to extract 129 features from lesion images. They then compared the recognition accuracy of three machine learning algorithms: support vector machine (SVM), random forest (RF) and K-nearest neighbor methods. Among them, the optimal SVM model achieved a recognition accuracy of 94.74% on the test set (Patil et al., 2017). employed two methods to extract features. The first method involved calculating HSV color moments, including mean, variance, skewness, energy, and entropy for each color channel. The second method utilized 6th-order Exact Legendre Moments. The multi-class SVM they proposed achieved an accuracy of 99.1% on a three-class tomato dataset (Meenakshi et al., 2019). focused their research on potato leaf images to evaluate the effectiveness of various methods for recognizing potato diseases. They compared traditional machine learning techniques with neural networks and found that the artificial neural network achieved a 92% recognition accuracy, significantly outperforming traditional methods like SVM and RF. Despite the simplicity of these machine learning algorithms themselves, manually extracting features is a highly complex process that often requires domain expert knowledge and a significant investment of time. The scale of data that can be processed is very limited (Liu et al., 2024). In recent years, with the continuous development of deep learning technology, research on the application of deep learning in plant disease recognition has been increasing (Rangarajan et al., 2018). used deep learning models, AlexNet and VGG16, which were pre-trained on ImageNet, to classify seven types of tomato leaf disease images in the dataset, achieving accuracy rates of 97.29% and 97.49%, respectively (Edna Chebet et al., 2019). compared state-of-the-art deep learning models for plant disease detection, including VGG16, ResNet50, and DenseNet. They observed that models with greater depth achieved higher accuracy. Among these models, the DenseNet model with 121 layers performed the best, achieving an accuracy of 99.75%. Zhao et al. (2021) integrated the multi-scale feature extraction module and SE module into ResNet50, significantly enhancing its feature extraction capability and achieving a recognition accuracy of 96.81% on the tomato leaf dataset. Zhou et al. (2021) restructured RDN model for classification task and achieve 95% recognition accuracy on tomato dataset (Liang and Jiang, 2023). proposed the ResNet50-DPA model, where cascaded atrous convolution and a dual-path attention mechanism were introduced to obtain features with different scales and to capture key features, respectively. However, in these studies, the high recognition accuracy often depends on deeper network structures, which usually have a large number of parameters and significant memory consumption, making them unsuitable for deployment on resource-constrained small edge devices (Choudhary et al., 2020).

**Figure 1:**
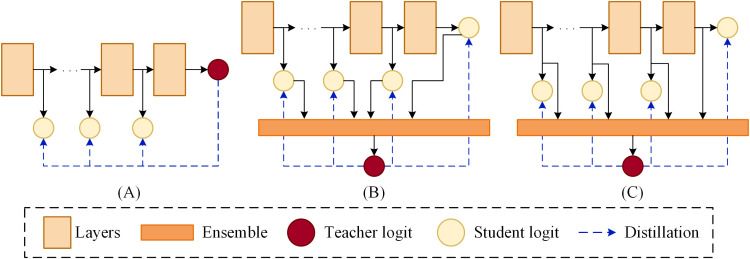
Schematic framework of distillation. **(A)** Self-distillation. **(B)** Averaged logits in self-distillation. **(C)** Ensemble self-distillation.

To address these challenges, We propose an ensemble self-distillation method and apply it to the lightweight model ShuffleNetV2, enabling its performance to reach the level of larger models. Unlike traditional knowledge distillation methods (Hinton et al., 2015), which rely on one-to-one knowledge transfer from a pre-trained teacher model to a student model, the approach proposed in this paper introduces a teacher model that is an ensemble of multiple student models. The knowledge of this teacher model is dynamically generated during the training process, avoiding the introduction of additional training costs. In contrast to traditional self-distillation methods, which typically use the original model as the teacher in the framework (as shown in **Figure 1A**), our method employs an ensemble of the original model and several shallow models as the teacher. This ensemble model is capable of fully integrating the information from each model, thereby providing the student models with richer and more comprehensive knowledge. **Figure 1B** illustrates a simple ensemble strategy where the logits from the original model and shallow models are averaged (Zhang et al., 2022) However, this approach neglects the intermediate feature maps from the individual models. Compared to deep feature maps, intermediate feature maps often contain more comprehensive information. Therefore, we further improve the ensemble strategy by integrating the intermediate feature maps from each model and performing deeper feature extraction (as shown in **Figure 1C**). In this way, the intermediate feature information from all branches is fully preserved for the ensemble model, allowing the ensemble model to utilize it more effectively to enhance its performance.

Specifically, we select ShuffleNetV2 as the student model. Based on its architecture, we build three shallow models at different depths to establish distillation framework. Each shallow models is equipped with unique structures and parameter sizes, mitigating the branch homogeneity issue commonly observed in traditional online knowledge distillation (Gong et al., 2023). After constructing these shallow models, we fuse the intermediate feature maps from each model. Based on this fused feature map, depthwise separable convolution layers are incorporated to further extract features, improving the performance of the ensemble model without a significant increase in parameter count. Once the distillation framework is established, two regularization terms are introduced: First, the Kullback-Leibler (KL) divergence is applied to constrain the logits of the student models, aligning them more closely with the outputs of the teacher model. Second, the L2 norm (Euclidean distance) is used to regulate the intermediate features of the student models, ensuring greater consistency with those of the teacher model.

The main contributions of this paper are summarized as follows:

1. We propose an ensemble method that fuses intermediate feature maps of all models within the distillation framework and incorporates depthwise separable convolution layers on the fused feature maps to further extract features, thereby constructing a more effective ensemble model.
2. We utilize the more effective ensemble model as a teacher to dynamically transfer knowledge to each student model during training. As a student model, the optimized ShuffleNetV2, namely KD-ShuffleNetV2, achieves performance comparable to larger models such as VGG16 and ResNet18, without altering the original architecture, making it more suitable for real-time tomato disease recognition on edge devices.
3. The shallow models within the framework can be treated as compressed versions of ShuffleNetV2, achieving different levels of compression in terms of parameter count and floating-point operations, providing flexibility for model deployment.

The rest of this paper is organized as follows: Section 2 introduces the dataset used and the data processing procedure, and provides a detailed description of the proposed distillation method. Section 3 presents various experiments conducted on the proposed method, analyzes the experimental results, and visualizes the model. Section 4 provides a summary of the paper and a discussion on its limitations.

## 2 Materials and methods

### 2.1 Data processing

#### 2.1.1 Image datasets

**Figure 2:**
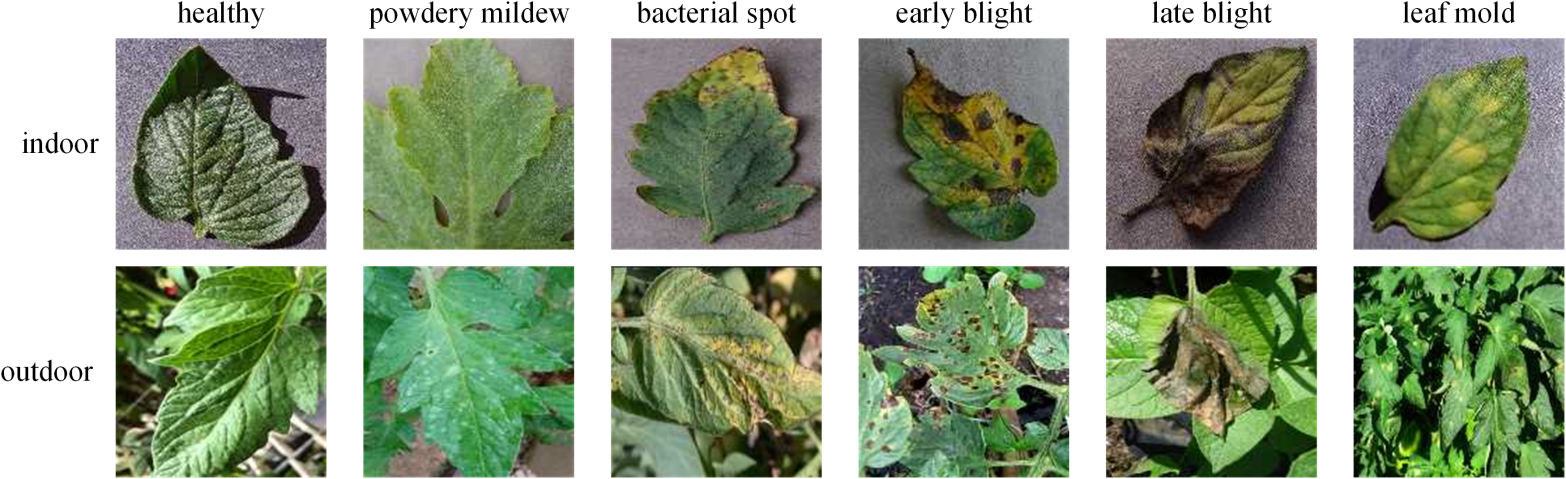
Examples of tomato diseases from the datasets.

The dataset used in this study is aggregated from four sources. The first source, Plant Village (SpMohanty, 2018), provides data samples on tomato leaf diseases from the following 10 categories: healthy(1591), bacterial spot(2127), early blight(1000), late blight(1909), leaf mold(952), septoria leaf spot(1771), yellow leaf curl virus(5357), mosaic virus(373), two-spotted spider mite(1676), and target spot(1404). The second source, Ai Challenger 2018 Crop Leaf Disease Challenge (Dataset AI Challenger, 2018), contains rich data samples on crop leaf diseases. However, we only use 1,469 samples of the powdery mildew category to fill the missing category in the Plant Village. The third source is PlantDoc (Singh et al., 2020), containing data samples of the following categories: healthy(62), bacterial spot(106), early blight(88), late blight(111), leaf mold(90), septoria leaf spot(155), yellow leaf curl virus(84), mosaic virus(54). The fourth source is Taiwan Tomato Disease (Huang and Chang, 2020), which contains data samples categorized as healthy(106), bacterial spot(110), late blight(98), leaf mold(67), powdery mildew(157), gray spot(84). The third and fourth sources contain abund rich ant outdoor samples, which further enhance the diversity of the our dataset. **Figure 2** shows examples of different tomato leaf diseases.

#### 2.1.2 Image preprocessing

The number of samples in the "gray spot" category within the Taiwan Tomato Disease is only 84, which is significantly lower than the number of samples in other categories in the aggregated dataset. Therefore, we excluded the "gray spot" category to achieve a more balanced distribution across categories. On one hand, there is a considerable imbalance in the number of samples across indoor categories. For instance, the mosaic virus, which has the fewest samples, consists of 373 instances, while the yellow leaf curl virus, the category with the most samples, contains 5,357 instances. On the other hand, the total number of indoor samples significantly exceeds that of outdoor samples. To mitigate this imbalance, random sampling was employed for the indoor categories with larger sample sizes, aiming to better balance the distribution both within the indoor categories and between indoor and outdoor data. **Table 1** summarizes the detailed information of the processed dataset used in this study. It should be noted that there are no outdoor samples available for two-spotted spider mite and target spot categories.

**Table 1:** Summary of main datasets used in the study.

Dataset	Plant Village		AI Challenger 2018		PlantDoc		Taiwan		Total
Class	Indoor	Outdoor	Indoor	Outdoor	Indoor	Outdoor	Indoor	Outdoor	
bacterial spot	1400				14	96	94	16	1620
early blight	1000				6	82			1088
healthy	1100				24	28		106	1258
late blight	1200				20	91	40	58	1409
leaf mold	952				6	85	63	4	1110
powdery dildew			1200				18	139	1357
septoria leaf spot	1150				29	128			1307
two-spotted spider mite	1676								1676
target spot	1404								1404
mosaic virus	373				6	48			427
yellow leaf curl virus	1900				7	231			2138
Total	12155		1200		112	789	215	323	14794

**Figure 3:**
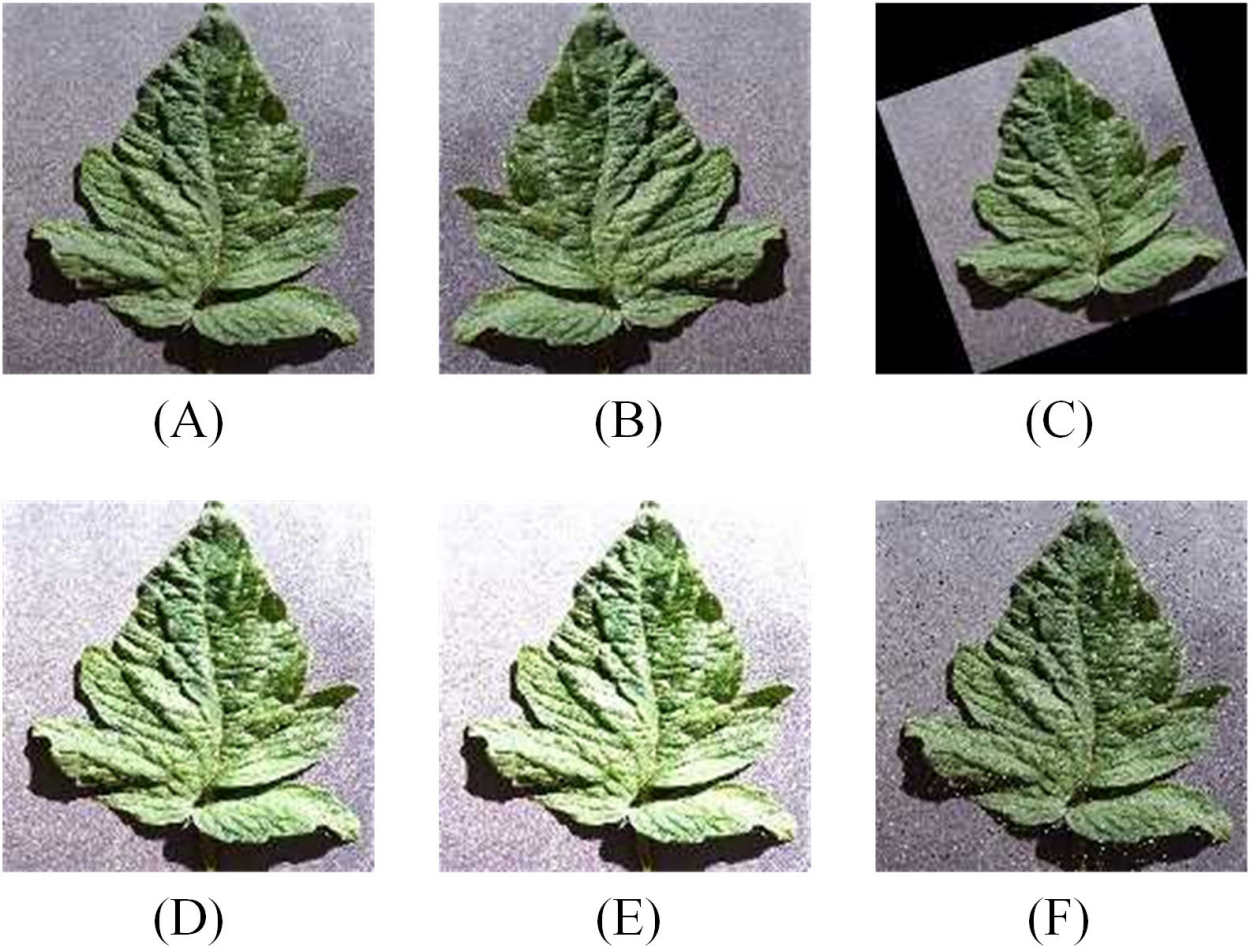
Data augmentation for training set **(A)** Original image. **(B)** Horizontal flip. **(C)** Random Rotate. **(D)** Brightness change. **(E)** Contrast change. **(F)** Add noise.

The data is resized to 64×64, and the entire dataset is split into training and testing sets in a 7:3 ratio. To enhance the diversity of the dataset, improving the model's generalization ability, data augmentation techniques, such as horizontal flipping, random rotation, brightness enhancement, contrast enhancement, etc., are applied exclusively to the training set during the model training phase. The effects of data augmentation are illustrated in **Figure 3**. The impact of data augmentation on the model's performance on the test set will be discussed in section 3.4.

### 2.2 The proposed method

#### 2.2.1 ShuffleNetV2 model

**Figure 4:**
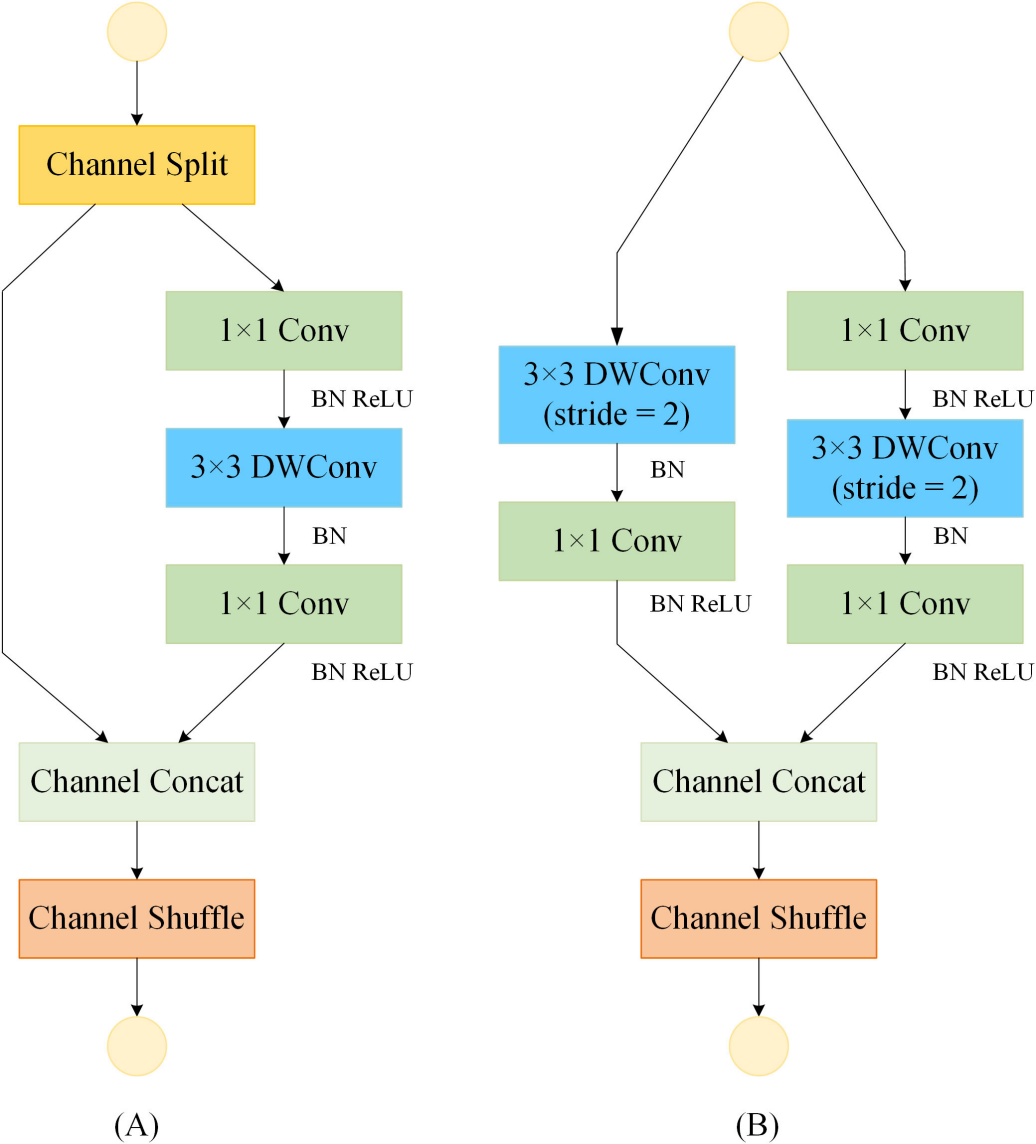
Basic feature extraction module for the ShuffleNetV2 model. "Conv" denotes standard convolution; "BN" denotes batch normalization; "ReLU" denotes activation function. **(A)** ShuffleNet Unit (SNU). **(B)** Downsample ShuffleNet Unit (D-SNU).

Ma et al. (2018) introduced a more lightweight ShuffleNetV2 unit, building on the ShuffleNetV1 architecture (Zhang et al., 2018), as depicted in **Figure 4**. The ShuffleNetV2 unit encompasses two variants: the ShuffleNet Unit (SNU) and the Downsample ShuffleNet Unit (D-SNU). The SNU, illustrated in **Figure 4A**, divides the input feature map channels into two branches, where the left branch remains unaltered, and the right branch employs a 1×1 standard convolution followed by a 3×3 depthwise convolution, concluding with another 1×1 standard convolution. In contrast, the D-SNU, shown in **Figure 4B**, directly partitions the input feature map channels into two branches, with the left branch incorporating a 3×3 depthwise convolution with a stride of 2, succeeded by a 1×1 standard convolution; the right branch mirrors this stride adjustment for its 3×3 depthwise convolution. This architecture effectively leverages the device's parallel processing capabilities and greatly minimizes computational costs. By shuffling the feature channels, it enables interaction between different channels, thereby improving the model's feature representation and reducing its overall complexity.

#### 2.2.2 Ensemble self-distillation framework

**Figure 5:**
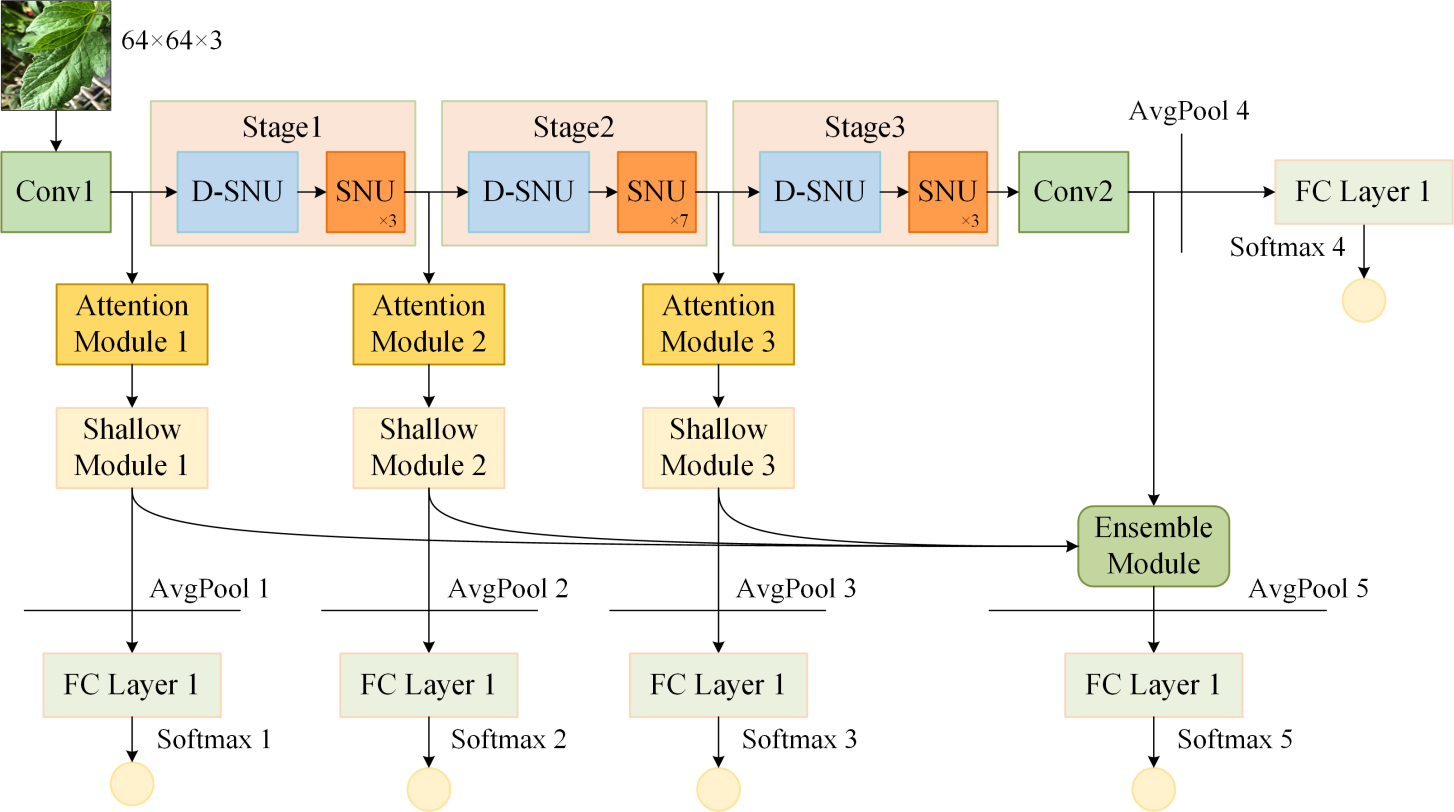
Ensemble self-distillation framework based on ShuffleNetV2. "AvgPool" denotes average pooling layer; "FC Layer" denotes fully connected layer; "Softmax" denotes softmax activation function.

The framework of ensemble self-distillation based on ShuffleNetV2 1.0x is constructed as illustrated in **Figure 5**. Conv1, positioned at the start of the ShuffleNetV2 and served to extract initial features from the input data, consists of 3×3 standard convolution and BN, while Conv2, positioned at the end of the ShuffleNetV2, consists of 1×1 standard convolution, BN, and ReLU. The ShuffleNetV2 is composed of Conv1, Stage 1, Stage 2, Stage 3, Conv2, and FC Layer 4. Each Stage is composed of one D-SNU and multiple SNUs.

Based on the structure and depth of the ShuffleNetV2, shallow models 1, 2, and 3 are constructed after Conv1, Stage 1, and Stage 2, respectively. The structure of shallow model 1 consists of Conv1, Attention Module 1, Shallow Module 1, and FC Layer 1. The structure of shallow model 2 consists of Conv1, Stage 1, Attention Module 2, Shallow Module 2, and FC Layer 2. The structure of shallow model 3 consists of Conv1, Stage 1, Stage 2, Attention Module 3, Shallow Module 3, and FC Layer 3. The deep feature maps from ShuffleNetV2 and the three shallow models are fused to construct the ensemble model. The ensemble model consists of an Ensemble Module and FC Layer 5. Before each FC Layer, an average pooling layer is placed to pool the input feature map to a size of 1×1. Subsequently, the output of each FC Layer is processed by the softmax function.

#### 2.2.3 Lightweight convolution structure

The architecture of the shallow model impacts not only the number of parameters in the ensemble self-distillation framework, which subsequently influences the overall training time, but also the efficiency of knowledge transfer. By treating the Attention Module and Shallow Module collectively as a projector that aligns shallow features with deep features, the parameter count of this projector plays a crucial role in determining the effectiveness of knowledge distillation (Chen et al., 2022). For the sake of lightweight design, both the Attention Module and the Shallow Module within the shallow model are built based on a Lightweight Convolution Structure (LCS) (Zhang et al., 2022). In this structure, the input feature map has a channel count of *in_c*, the output feature map has a channel count of *out_c*, and the stride is denoted as *s*. This is represented as LCS(*in_c, out_c, s*).

**Figure 6:**
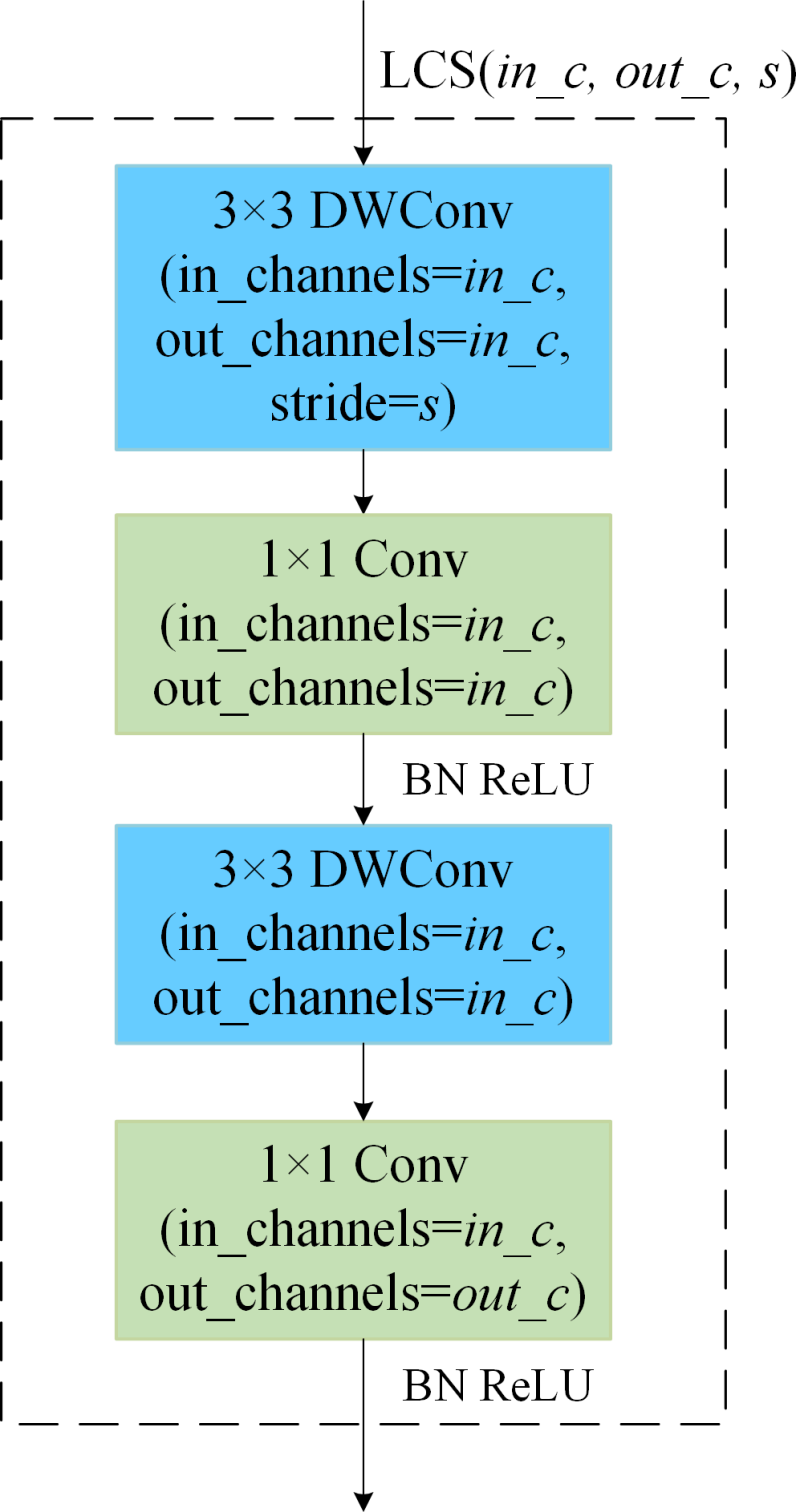
Lightweight convolution structure.

As shown in **Figure 6**, the LCS(*in_c, out_c, s*) structure consists of two groups of depthwise separable convolutions. Each group of depthwise separable convolutions is composed of a 3×3 depthwise convolution and a 1×1 pointwise convolution. The depthwise convolution in the first group has a stride of *s*, while the stride for all other convolution operations is set to 1 by default.

#### 2.2.4 Attention module

**Figure 7:**
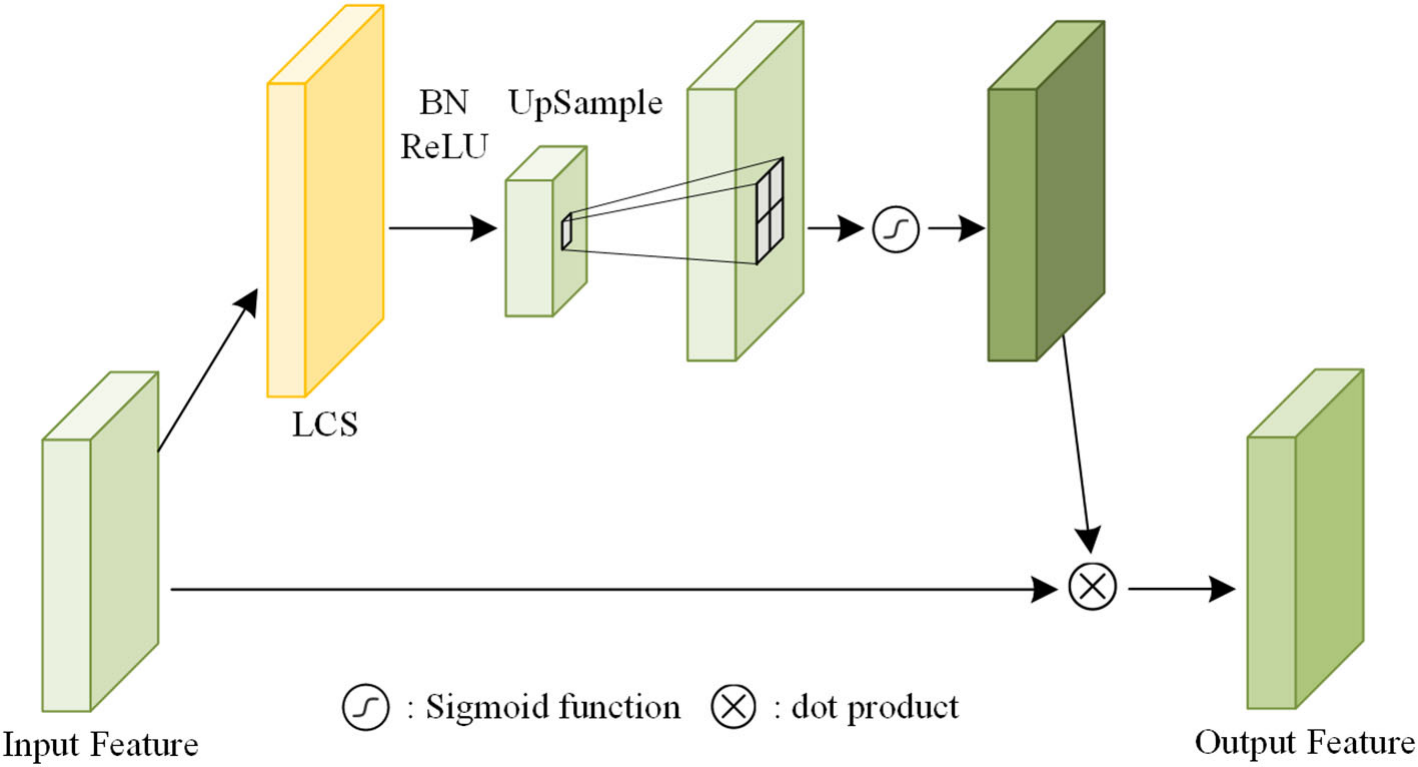
Attention module.

To decide which shallow features are distilled, the Attention Module, as illustrated in **Figure 7**, is introduced. The input feature map is first processed by a LCS with a stride of 2, reducing the spatial resolution through downsampling. Subsequently, an upsampling layer using bilinear interpolation with a scaling factor of 2 is applied to restore the original resolution. A sigmoid activation function is then used to generate the attention mask. Finally, the attention mask is element-wise multiplied with the input feature map to obtain the output feature map (Zhang et al., 2022). In detail, the LCS configurations corresponding to Attention Modules *i* (where *i*=1, 2, 3) are LCS(24,24,2), LCS(116,116,2), and LCS(232,232,2), respectively.

#### 2.2.5 Shallow module

The architecture of the Shallow Module is constructed by stacking multiple LCS in sequence (Zhang et al., 2022). When designing the Shallow Module, the number of stacked LCSs and the size of (*in_c, out_c*) can be adjusted to ensure that, in terms of the number of parameters, the shallow model 1 is less than shallow model 2, and shallow model 2 is less than shallow model 3 (i.e., shallow model 1< shallow model 2< shallow model 3), so as to build a hierarchical structure and avoid the problem of homogenization of the models (Gong et al., 2023). Specifically, Shallow Module 1 consists of LCS(24, 116, 2), LCS(116, 464, 2), and LCS(464, 1024, 2); Shallow Module 2 is formed by stacking LCS(116, 464, 2) and LCS(464, 1024, 2); and Shallow Module 3 is composed of LCS(232, 464, 2) followed by LCS(464, 1024, 1).

#### 2.2.6 Ensemble module

**Figure 8:**
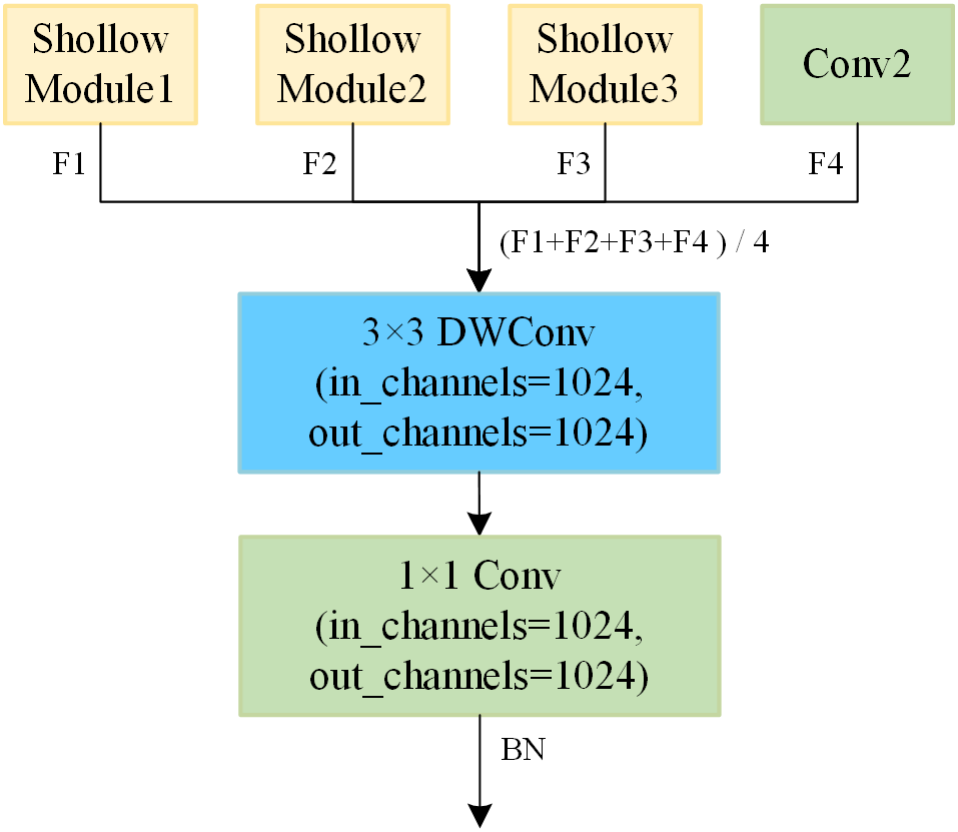
Ensemble module.

The shape of feature maps in the original model and shallow model are shown in **Table 2**. It can be observed that the output feature maps of Shallow Modules *i* (where *i*=1, 2, 3) and Conv2 have the same shape, all being 8×8×1024. Consequently, the four feature maps are fused into a single feature map through averaging, which serves as the input feature map for the ensemble module. The fused feature maps exhibit a higher level of redundancy, leading to insufficient feature extraction. At the same time, employing average pooling followed by classification with fully connected layers results in significant information loss. To address these issues, we apply further convolution to the fused feature maps to construct a more robust ensemble model. As illustrated in the **Figure 8**, the ensemble module is composed of 3×3 depthwise convolution, 1×1 standard convolution, and BN, which further extracts features based on the fused feature maps. The decision to omit the ReLU activation function is based on the fact that nonlinearity has already been introduced in each branch, and since the ensemble module is positioned deeper in the model, the use of ReLU could lead to further information loss. Other ensemble methods will be discussed in Section 3.3.1.

**Table 2:** Layer structure and corresponding feature map shape.

Layer		Feature map shape			
		shallow model 1 (*i*=1)	shallow model 2 (*i*=2)	shallow model 3 (*i*=3)	ShuffleNetV2 (*i*=4)
Input		64×64×3	64×64×3	64×64×3	64×64×3
Conv1		64×64×24	64×64×24	64×64×24	64×64×24
Attention Module1		64×64×24	–	–	–
Shallow Module1	LCS(24,116,2)	32×32×116	–	–	–
	LCS(116,464,2)	16×16×464	–	–	–
	LCS(464,1024,2)	8×8×1024	–	–	–
Stage1		–	32×32×116	32×32×116	32×32×116
Attention Module2		–	32×32×116	–	–
Shallow Module2	LCS(116,464,2)	–	16×16×464	–	–
	LCS(464,1024,2)	–	8×8×1024	–	–
Stage2		–	–	16×16×232	16×16×232
Attention Module3		–	–	16×16×232	–
Shallow Module3	LCS(232,464,2)	–	–	8×8×464	–
	LCS(464,1024,1)	–	–	8×8×1024	–
Stage3		–	–	–	8×8×464
Conv2		–	–	–	8×8×1024
AvgPool *i*		1×1×1024	1×1×1024	1×1×1024	1×1×1024

#### 2.2.7 Loss function

Given a training sample *x* and an one-hot true label *y*, we can get the logit output $z_{i}\in {\mathbb{R}}^{1\times C}$ (where *i* = 1, 2, 3, 4, 5), where each *z_i_* represents the outputs of corresponding FC Layers mentioned above, *C* is the number of classes. By knowledge distillation method, we can acquire the final prediction after a softmax layer:


(1) $$p_{i,j,T}=\frac{\exp(\frac{z_{i,j}}{T})}{\sum_{c=1}^{C}\exp(\frac{z_{i,c}}{T})}$$

where *T* denotes the hyperparameter temperature, *z_i,j_* represents the logit of the *j*-th class of the *i*-th logit, *p_i,j,T_* represents the probability of the *j*-th class of the *i*-th logit at temperature *T*. In a certain range, as *T* increases, the model's predictions become smoother, and the inherent 'dark knowledge' (Hinton et al., 2015) becomes richer. When *T* = 1.0, the output will become to vanilla softmax output. By introducing KL divergence loss during distillation training, the student model can learn the 'dark knowledge' from the teacher model, thereby enhancing the overall performance of the student model.

The proposed method employs an ensemble model as the teacher, while ShuffleNetV2 and each shallow model serving as students for distillation training. This approach incorporates three losses: *L*_ce_, *L*_kd_, and *L*_fkd_. Thus, the overall objective *L*_total_ is formulated as:


(2) $$L_{\text{total}}=L_{\text{ce}}+L_{\text{kd}}+L_{\text{fkd}}$$

*L*_ce_ represents the cross-entropy loss between the ensemble model, the ShuffleNetV2 model, each shallow model, and the ground truth labels of the dataset, as defined in Equation 3. This loss function ensures that the ensemble model, the ShuffleNetV2 model, and each shallow model are all trained under the supervision of the ground truth labels from the dataset.


(3) $$L_{\text{ce}}=CE(p_{i=5,T=1},y)+\left(1-\alpha \right)\cdot \sum_{i=1}^{4}CE(p_{i,T=1},y)$$

where CE represents the cross-entropy loss function, and α is a hyperparameter to balance the weight of the student models' cross-entropy loss and the logit distillation loss.

*L*_kd_ represents the KL divergence loss between the ShuffleNetV2 model, each shallow model, and the ensemble model, as shown in Equation 4. This loss allows the output of the ensemble model to serve as the learning target for both the ShuffleNetV2 model and the shallow model.


(4) $$L_{\text{kd}}=\alpha \cdot \sum_{i=1}^{4}KL(p_{i,T=3},p_{i=5,T=3})$$

*L*_fkd_ represents the L2 loss calculated between the feature outputs of the ensemble model and those of shallow models 1 and 2, as shown in Equation 5. This loss aims to align the features of shallow models 1 and 2 with those of the ensemble model, enabling the shallower models to directly learn the features of the deeper model, thereby enhancing the effectiveness of the distillation training.


(5) $$L_{\text{fkd}}=\beta \cdot \sum_{i=1}^{2}||H_{i}-H_{5}{||}_{2}^{2}$$

where each *H_i_* (for *i* = 1, 2, 3, 4, 5) represents the outputs of corresponding AvgPool layers mentioned above, and β is a scaling factor to control the magnitude of the feature distillation loss.

## 3 Results and discussion

In this section, we provide a detailed description of the experimental setup, evaluation metrics, and results. Additionally, we discuss various ensemble methods, the selection of hyperparameters, and comparisons with other models. It should be noted that, to distinguish the version integrated into the ensemble self-distillation framework from the original ShuffleNetV2, we refer to it as KD-ShuffleNetV2. The architecture of KD-ShuffleNetV2 is identical to the original ShuffleNetV2, which means that they have the same number of parameters and floating-point operations (FLOPs). The only difference is that KD-ShuffleNetV2 is optimized by the proposed distillation method to have higher recognition accuracy without changing its architecture.

### 3.1 Experimental setup

The hardware used in this experiment includes an Intel^®^ Xeon^®^ CPU and an Nvidia Tesla P100 16G GPU. The operating system is Linux 5.15.133+, and the required tool versions are Python 3.10.13, PyTorch 2.1.2, and CUDA 12.1. The scikit-learn library was used to calculate the evaluation indictors mentioned in section 3.2. The ptflops library is utilized to compute the number of parameters and floating-point operations (FLOPs).

The parameters used in the experiment will affect the experimental results, and the values of each parameter are summarized in **Table 3**. The model is trained for 100 epochs with a batch size of 128. The initial learning rate is set to 0.01 and decayed by 30% every 10 epochs. The parameters are updated using the stochastic gradient descent (SGD) optimizer with a weight decay of 5×10^-4^ and a momentum of 0.9. The hyperparameters α and β are set to 0.1 and 5×10^-4^, respectively. Distillation temperature *T* is set to 3.0.

**Table 3:** Parameter value.

Parameter	Value
Batch size	128
Image size	64×64
Optimization algorithm	SGD
Initial learning rate	0.01
Number of epochs	100
α	0.1
β	5×10-4
T	3.0

### 3.2 Evaluation indictors

To evaluate the performance of the models, this paper considers and number of parameters and computer as the evaluation criteria for model complexity. For evaluating model performance, accuracy, precision, recall, and F1 score on the test set are used as the primary indicators. The calculation methods for these four performance metrics are shown in Equations 6–9.


(6) $$\text{Accuracy = }\frac{\text{TP+TN}}{\text{TP+TN+FP+FN}}$$

(7) $$\text{Precision}=\frac{\text{TP}}{\text{TP}+\text{FP}}$$

(8) $$\text{Recall}=\frac{\text{TP}}{\text{TP}+\text{FN}}$$

(9) $$\text{F}1=\frac{2\times \text{Precision}\times \text{Recall}}{\text{Precision}+\text{Recall}}$$

Where TP is the result of correctly predicting positive classification; FP is the result of incorrectly prediction of positive classification; TN is the result of correctly predicting negative classification; FN is the result of incorrectly predicting negative classification.

After data preprocessing, the dataset in this study exhibits a relatively balanced distribution across categories, but there are still some categories with fewer samples compared to others. In cases of class imbalance, directly using unweighted metrics can lead to evaluation results that are biased toward the categories with larger sample sizes, potentially neglecting the performance of those categories with fewer samples. To provide a more comprehensive assessment of the model's performance, we used the scikit-learn library to compute precision, recall, and F1 score in a weighted average manner.

### 3.3 Model performance comparison

#### 3.3.1 Discussion of different ensemble modules

In this section, we introduce four additional ensemble methods: Avg (Zhang et al., 2018), Concat (Wu and Gong, 2021), Naive (Guo et al., 2020), and MinLogit (Guo et al., 2020). We only adopt the ensemble ideas from these methods and use them as benchmarks to evaluate the effectiveness of the proposed approach.

1. Avg method: A widely adopted standard, this method computes the average of the logits from all branches to form the ensemble model's logits.
2. Concat method: This method concatenates the logits from all branches along the channel dimension, preserving the information from each branch. Then, a fully connected layer is applied for training ensemble model.
3. Naive method: In this method, the logit with the lowest cross-entropy loss with respect to the true label is selected from all logits across branches. This selected logit serves as the teacher for all students.
4. MinLogit method: The MinLogit method selects the minimum value at each corresponding position across the logits to form the ensemble model's logits, aiming to minimize the cross-entropy loss between the ensemble's predictions and the true labels.

To improve the clarity of our experimental results, we focus exclusively on the accuracy of KD-ShuffleNetV2 and the ensemble model on the test set. The experimental results are shown in **Table 4**. In both the Avg and MinLogit methods, the accuracy of the ensemble model did not exhibit a significantly higher level compared to the accuracy of the KD-ShuffleNetV2, which limits the teacher's capacity to transfer generalized knowledge to the student, thereby hindering the effective improvement of the student's model performance. In the Naive and Concat methods, the accuracy of the ensemble model is significantly higher than that of KD-ShuffleNetV2, but it still fails to effectively improve the accuracy of KD-ShuffleNetV2. In contrast, compared to all other ensemble schemes, the proposed approach offers a superior ensemble model, achieving an accuracy of 95.15% on the test set. This high-performance ensemble model also effectively transfers knowledge to KD-ShuffleNetV2. As a result, the KD-ShuffleNetV2 optimized by the proposed method achieves an accuracy of 95.08%, surpassing other approaches.

**Table 4:** Results of different ensemble methods for KD-ShuffleNetV2 and Ensemble model.

Model	Avg	Concat	Naive	MinLogit	Ours
KD-ShuffleNetV2	94.52	94.59	94.57	94.63	95.08
Ensemble model	94.61	94.86	95.04	94.63	95.15

#### 3.3.2 Comparison of results for different models

To further verify the effectiveness of the KD-ShuffleNetV2 and other models within the ensemble self-distillation framework, this paper compared them with the Vgg16 (Simonyan and Zisserman, 2014), ResNet18 (He et al., 2016), MobileNetV1 (Howard et al., 2017), MobileNetV2 (Sandler et al., 2018), MobileNetV3 (Howard et al., 2019) and MobileVit (Mehta and Rastegari, 2021) models under the same test conditions. The experimental results are presented in **Table 5**. Compared to the original ShuffleNetV2, KD-ShuffleNetV2 achieves significant improvements in accuracy, precision, recall, and F1-score, with respective gains of 1.35%, 1.36%, 1.37%, and 1.37%; Shallow Model 3 reaches an accuracy of 95.04%, representing a 1.31% increase, while maintaining a negligible change in parameter count and reducing FLOPs by 6.01%; Shallow Model 2 achieves an accuracy of 94.30%, demonstrating a 0.57% improvement, while reducing parameters by 33.71% and FLOPs by 42.08%, making it a promising choice for resource-limited environments; Shallow Model 1 achieves an accuracy of 93.21%, with a slight decrease of 0.52%, but offers substantial reductions of 37.77% in parameters and 60.11% in FLOPs. The ensemble model achieves the highest accuracy of 95.15%, with a 1.42% improvement, making it more suitable for scenarios where accuracy is prioritized, and deployment resources are abundant.

**Table 5:** Results of different models on the test set.

Model	Accuracy/%	Precision/%	Recall/%	F1 score/%	Params	FLOPs
Vgg16	94.27	93.79	93.76	93.75	33650763	1.28×10^9^
ResNet18	94.57	94.14	94.007	94.07	11271432	2.23×10^9^
MobileNetV3_Small	92.29	91.74	91.68	91.67	1529131	5.84×10^6^
MobileNetV3_Large	92.51	92.00	91.92	91.91	4216123	2.13×10^7^
MobileVit_XS	93.01	92.57	92.55	92.53	2003168	5.72×10^7^
MobileVit_S	93.03	92.47	92.41	92.40	5003760	1.10×10^8^
MobileNetV1	92.33	91.92	91.85	91.83	3224203	1.93×10^8^
MobileNetV2	94.14	93.65	93.55	93.51	2255371	2.28×10^8^
ShuffleNetV2	93.73	93.22	93.18	93.17	1269671	1.83×10^8^
KD-ShuffleNetV2	95.08	94.58	94.55	94.54	1269671	1.83×10^8^
Shallow model3	95.04	94.44	94.38	94.38	1268890	1.72×10^8^
Shallow model2	94.30	93.75	93.71	93.70	841660	1.06×10^8^
Shallow model1	93.21	92.61	92.50	92.49	790090	7.31×10^7^
Ensemble model	95.15	94.65	94.63	94.61	4936699	4.27×10^8^

Compared to VGG16, the ensemble model and KD-ShuffleNetV2 improve accuracy by 0.88% and 0.81%, respectively, while reducing parameters by 85.33% and 96.23%, and FLOPs by 66.64% and 85.70%, respectively. Similarly, compared to ResNet18, the ensemble model and KD-ShuffleNetV2 achieve accuracy improvements of 0.58% and 0.51%, respectively, while reducing parameters by 56.20% and 88.74%, and FLOPs by 80.85% and 91.79%.

When compared to other listed lightweight models, KD-ShuffleNetV2 and shallow models demonstrate superior accuracy with relatively lower parameter counts. Specifically, compared to MobileNetV2, which achieves the highest accuracy among the listed lightweight models, KD-ShuffleNetV2, Shallow Model 3, and Shallow Model 2 achieve accuracy improvements of 0.94%, 0.90%, and 0.16%, respectively, while reducing parameter counts by 43.70%, 43.73%, and 62.68%, and FLOPs by 19.74%, 24.56%, and 53.51%, respectively. Compared to MobileViT_S, which has the largest parameter count among the lightweight models, KD-ShuffleNetV2 reduces parameters by 74.63% while improving accuracy by 2.05%. Similarly, Shallow Model 1 reduces parameters by 84.21% and improves accuracy by 0.18%.

**Figure 9:**
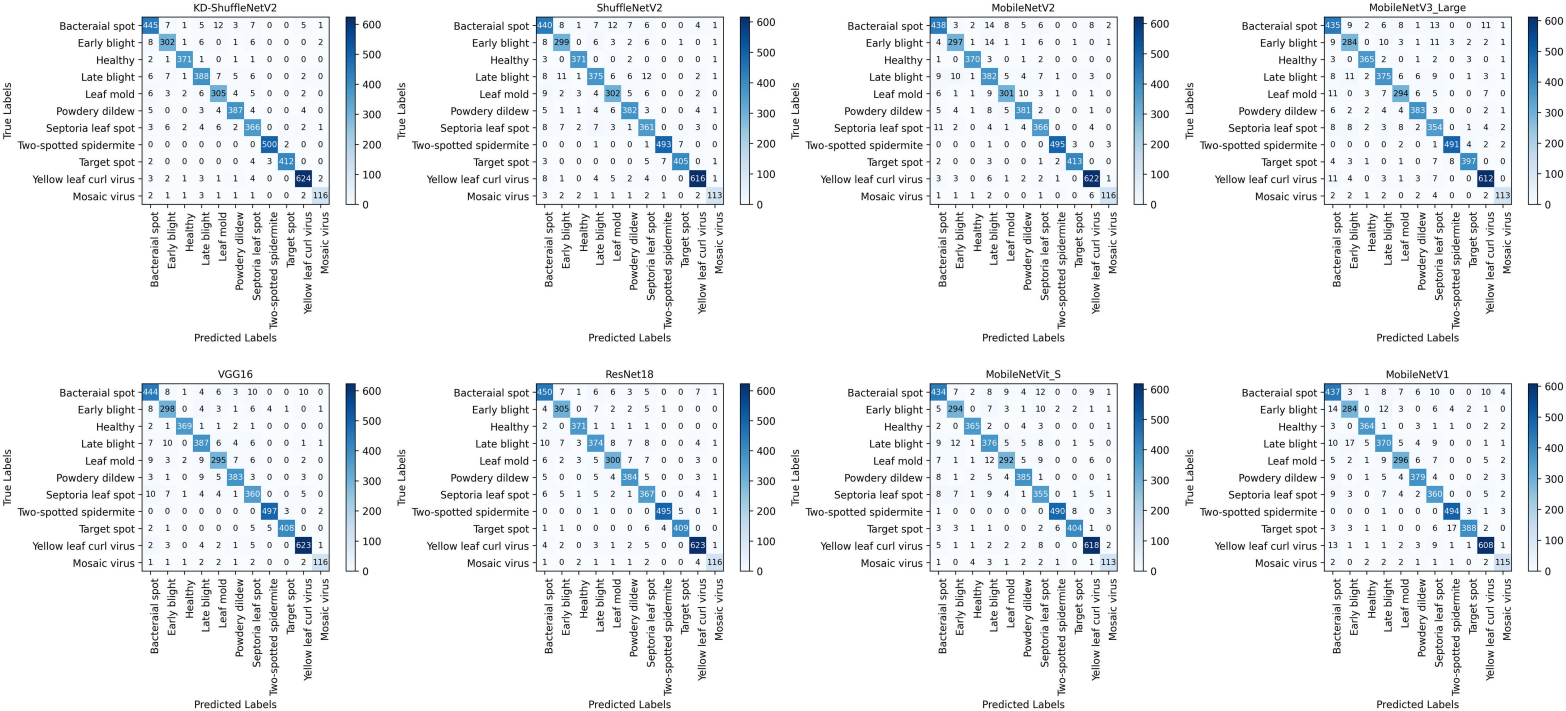
Confusion matrices of different models.

The classification results, as visualized in the confusion matrix in **Figure 9**, demonstrate the performance of the model. The vertical axis represents the 11 categories of tomato leaf diseases in the dataset, while the horizontal axis corresponds to the categories predicted by the model. Compared to the original ShuffleNetV2, except for the healthy category, KD-ShuffleNetV2 achieved a notable improvement in the number of correctly predicted samples across all categories. The most significant enhancement was observed in the late blight category, where an additional 13 samples were accurately classified.

### 3.4 Discussion on data augmentation

To investigate the impact of data augmentation on the generalization ability of models, we conducted experiments to evaluate the accuracy of each model on the test set without data augmentation and compared them with those using data augmentation. The experimental results are shown in **Table 6**. It can be observed that after data augmentation, the accuracy of all models on the test set improved. Without data augmentation, although the accuracy of KD-ShuffleNetV2 does not reach the level of larger models such as Vgg16 and ResNet18, it still surpasses all the lightweight models listed. Specifically, the accuracy of KD-ShuffleNetV2 (92.15%) is 1.44% higher than that of MobileNetV2 (90.71%). Additionally, compared to the original ShuffleNetV2, the accuracy of KD-ShuffleNetV2 improved by 3.29% without data augmentation and by 1.35% with data augmentation, demonstrating that the proposed method effectively enhances model performance regardless of the use of data augmentation.

**Table 6:** The impact of data augmentation on experimental results.

Model	Vgg16	ResNet18	MobileNetV3_Large	MobileVit_S	MobileNet V2	ShuffleNet V2	KD-ShuffleNetV2
without data augment	93.12	92.92	89.63	87.62	90.71	88.86	92.15
with data augment	94.27	94.57	92.51	93.03	94.14	93.73	95.08

### 3.5 Discussion on the hyperparameter

To verify the effectiveness of logit distillation and feature distillation, we conducted experiments with different values of α and β at a temperature of 3.0. First, by fixing the initial value of β at 1×10^-4^, we explored the impact of different α on the experimental results, as shown in **Table 7**. As α increased, the accuracy of both KD-ShuffleNetV2 and the ensemble model reached its peak at α = 0.1. Other models reached their peak slightly later, but also exhibited an increasing trend followed by a decrease. Subsequently, we fixed α at 0.1 and investigated the impact of different β on the experimental results. As shown in **Table 8**, as β increased, there was no clear trend in the accuracy of the models. However, except for the shallow model 2, the accuracy of all other models reached peak accuracy at β = 5×10^-4^.

**Table 7:** Comparison of models' accuracy under varying α values with fixed β =1×10^-4^.

Model	α					
	0	0.1	0.2	0.3	0.4	0.5
KD-ShuffleNetV2	94.63	94.90	94.86	94.61	94.63	94.50
Shallow model3	94.45	94.86	94.81	94.63	94.50	94.36
Shallow model2	94.36	94.30	94.43	94.32	94.18	93.98
Shallow model1	92.69	92.69	92.76	93.08	92.58	92.27
Ensemble model	94.93	94.97	95.04	94.81	94.84	94.68

**Table 8:** Comparison of models' accuracy under varying β values with fixed α =1×10^-4^.

Model	β					
	0	1×10^-4^	3×10^-4^	5×10^-4^	7×10^-4^	9×10^-4^
KD-ShuffleNetV2	94.72	94.90	94.66	95.08	94.66	94.45
Shallow model3	94.72	94.86	94.72	95.04	94.79	94.54
Shallow model2	94.25	94.30	94.50	94.30	94.48	94.30
Shallow model1	92.99	92.69	93.12	93.21	93.08	93.19
Ensemble model	95.08	94.97	95.04	95.15	94.84	94.88

When α = 0, the experiment corresponded to training without logit distillation, leading to a 0.45% decrease in accuracy compared to the optimal case with logit distillation for KD-ShuffleNetV2. Similarly, when β = 0, the experiment corresponded to training without feature distillation, resulting in a 0.36% accuracy drop compared to the optimal case with feature distillation for KD-ShuffleNetV2. This demonstrates that both distillation methods effectively transfer knowledge from the teacher model, and their combined use is essential for maintaining or enhancing the model's generalization ability.

### 3.6 Visualization of experimental results

**Figure 10:**
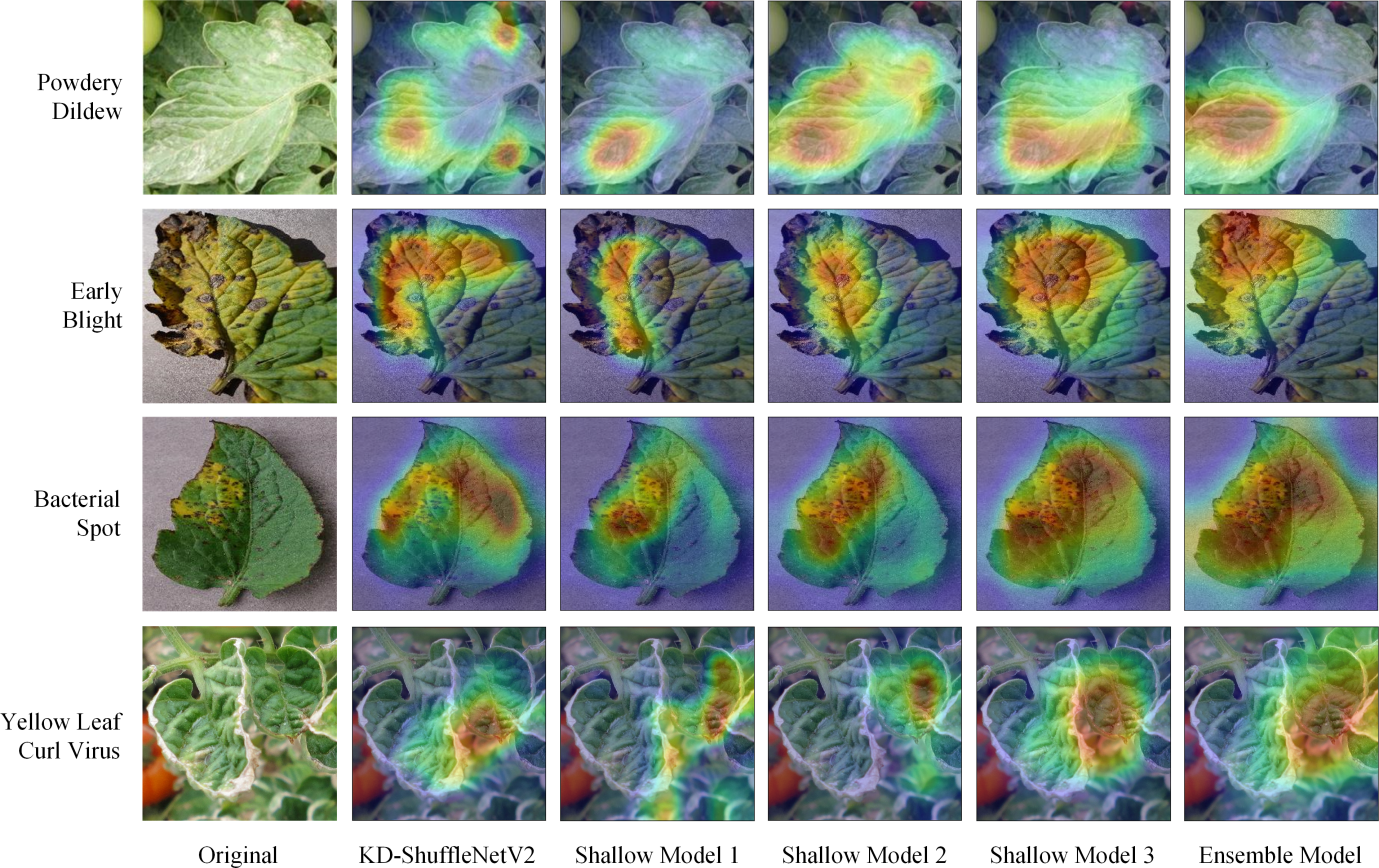
Heatmaps of all branches within the distillation framework.

The three shallow models in the ensemble self-distillation framework are built based on KD-ShuffleNetV2. KD-ShuffleNetV2 along with each shallow model can be viewed as different branches of the overall structure. To investigate the regions each branch focuses on during training, Grad-CAM is applied to visualize the heatmap for each branch's attention to the input data. As shown in **Figure 10**, it is easy to observe that, from the first to the third shallow model, the regions highlighted by Grad-CAM gradually expand, and the areas of focus for each branch differ significantly. This diversity in attention regions may contribute to the improvement of the ensemble model's performance. The ensemble model integrates information from all branches, resulting in a broader area of attention. Therefore, the ensemble model, as the teacher model, can provide more generalized knowledge to each branch. By analyzing the heatmaps of the powdery mildew samples, it can be observed that KD-ShuffleNetV2 accurately focuses on the disease-affected regions of the leaves, even though they are dispersed across different locations.

## 4 Conclusions

In order to address the challenge of deploying high-precision large models on edge devices for real-time tomato disease detection, we propose a method based on enesmble self-distillation. This method successfully improves the accuracy of KD-ShuffleNetV2, achieving not only the lowest parameter count among all the listed lightweight models but also the highest accuracy. Furthermore, its accuracy surpasses that of larger models like VGG16 and ResNet18, demonstrating the successful transfer of knowledge from the large model to the small model. The entire training process requires only one stage, significantly reducing the training cost compared to the two stages required by traditional knowledge distillation methods. In terms of creating the ensemble model, our proposed enesmble method effectively transfers knowledge to the student model, outperforming other methods, such as averaging logits. Moreover, heatmap results show that the multiple shallow models used to assist online knowledge distillation, as well as the KD-ShuffleNetV2, focus on different regions of the tomato leaf disease, enhancing the diversity of the branches and contributing to the improved performance of the ensemble model. Additionally, the multiple shallow models achieve varying levels of compression of original ShuffleNetV2. Compared to the original ShuffleNetV2, shallow model 2 improves accuracy by 0.57%, while reducing the parameter count and FLOPs by 33.71% and 42.08%, respectively. Despite these promising results, there are some limitations to our current framework. Although the proposed approach can be applied to any deep learning model, it requires customization for each specific model, which is often a complex process. Moreover, when applied to CNN models integrated with Transformers, such as MobileNetV3 and MobileVit, it often yields suboptimal results.

## Data Availability

The datasets presented in this study can be found in online repositories. The names of the repository/repositories and accession number(s) can be found below: https://github.com/AIChallenger/AI_Challenger_2018; https://github.com/spMohanty/PlantVillage-Dataset; https://doi.org/10.17632/ngdgg79rzb.1; https://doi.org/10.1145/3371158.3371196.
